# The mid-point transverse process to pleura (MTP) block in chest trauma: a game-changer

**DOI:** 10.1016/j.bjane.2021.04.019

**Published:** 2021-04-28

**Authors:** Manbir Kaur, Priyanka Sethi, Ravindra Singh, Pradeep Bhatia

**Affiliations:** All India Institute of Medical Sciences (AIIMS), Department of Anesthesia and Critical Care, Jodhpur, India

Dear Editor,

Road traffic accidents have emerged as a pandemic of the modern world. Chest trauma, being commonly associated with these road traffic accidents, becomes the leading cause of morbidity and mortality. Management of pain is essential in these trauma patients as, along with patient comfort, it also decreases patient’s respiratory complications.^1^ Various techniques are designed to reduce pain-related complications, including pharmacological drug therapies, and regional or neuraxial nerve blocks techniques. Thoracic Epidural Analgesia (TEA) is considered as gold standard for relieving rib fracture pain. However, it is not free of complications, including dural puncture, accidental hypotension, and cardiovascular collapse.^2^ Likewise, the thoracic paravertebral block might also lead to inadvertent vascular puncture, hypotension, epidural or intrathecal spread, pleural puncture, and pneumothorax.^3^

Mid-point transverse process to pleura (MTP) block is a recently described ultrasound-guided novel technique that involves injecting the drug at mid‐point between the transverse process and the pleura.^4^ In this block, a high frequency (8–15 MHz) linear ultrasound probe is placed obliquely approximately 3 cm laterally from the midpoint of the spinous process. The block needle (50 mm long) is advanced from caudal to cranial direction of the paravertebral space. When the needle tip reaches the midpoint between the transverse process and the pleura, the drug is given. The drug spreads to the dorsal and ventral rami in the paravertebral space through the fenestrations in the superior costotransverse ligament at the level of injection (Fig. 1).^4^ Being a superficial block, landmarks are quickly and easily felt.^5^ Thus, it is comparatively easy to insert. It can be easily applied to obese traumatic patients with a compromised position. Due to these advantages, MTP block is much safer than thoracic epidural and thoracic paravertebral blocks by minimizing the risk of pleural puncture and inadvertent intrathecal injections.

**Figure 1:**
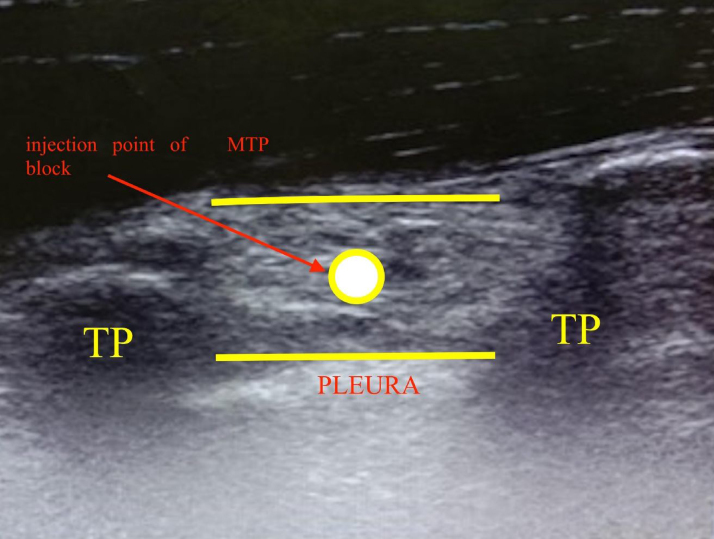
Depicts the ultrasound image of MTP block. The white circle represents the point of injection of MTP block. TP, transverse process; MTP, mid‐point transverse process to pleura.

Management of acute pain is highly advantageous for enhanced recovery after trauma to prevent the neuroendocrine stress response and thus combat the cascade of events occurring after activation of the sympathetic nervous system and catecholamine release. Therefore, we believe that MTP block can be a game-changer for the chest trauma patients due to an easy approach and reduced risk profile.

## Conflicts of interest

The authors declare no conflicts of interest.
